# Evaluating Species Delimitation Methods in *Chloroidium* (Trebouxiophyceae, Chlorophyta): Efficacy of DNA Barcodes and Description of *Chloroidium pseudoellipsoideum* sp. nov. from Arctic Soils

**DOI:** 10.3390/plants14243739

**Published:** 2025-12-08

**Authors:** Elena Krivina, Maria Sinetova, Alexander Starikov, Aleksey Portnov, Anna Temraleeva

**Affiliations:** 1 G.K. Skryabin Institute of Biochemistry and Physiology of Microorganisms, Pushchino Scientific Center for Biological Research of the Russian Academy of Sciences, 142290 Pushchino, Russia; temraleeva.anna@gmail.com; 2 K.A. Timiryazev Institute of Plant Physiology, Russian Academy of Sciences, 127276 Moscow, Russia; maria.sinetova@mail.ru (M.S.); starikovay1393@gmail.com (A.S.); 3 Institute of Physico-Chemical and Biological Problems in Soil Science, Pushchino Scientific Center for Biological Research of the Russian Academy of Sciences, 142290 Pushchino, Russia; alekseyportnow@gmail.com

**Keywords:** molecular phylogeny, DNA barcoding, species delimitation, fatty acid composition

## Abstract

Despite extensive research into green microalgae belonging to the genus *Chloroidium*, their species diversity and biotechnological potential remain poorly characterized. The strain VKM Al-418, the subject of this study, was isolated from the soil of Duvannyi Yar (Russian Federation). The independent species status of this strain is supported by distinct morphological characteristics, robust phylogenetic placement based on the 18S-ITS1-5.8S-ITS2 fragment, and unique features in the secondary structures of both ITS1 and ITS2, including one compensatory base change (CBC) in the highly conserved helix III of ITS2. Additionally, the species delimitation was also confirmed using five independent algorithmic approaches analyzing four different DNA barcodes. The concatenated ITS1-5.8S-ITS2 fragment is more reliable for species discrimination than the individual ITS1 or ITS2 barcodes. Of the species delimitation methods evaluated, ASAP (Assemble Species by Automatic Partitioning) and GMYC (Generalized Mixed Yule Coalescent) performed best in distinguishing *Chloroidium* species across multiple barcode regions in our analysis. The fatty acid profile of strain VKM Al-418 was analyzed at 9 °C, 22 °C, and 27 °C and exhibited high plasticity in response to temperature, indicative of an adaptive strategy to its harsh environment. Using this integrative taxonomic approach, we describe *Chloroidium pseudoellipsoideum* sp. nov., a new species with a distinct phylogenetic positioning and promising biotechnological properties.

## 1. Introduction

The genus *Chloroidium* includes coccoid green microalgae characterized by their ellipsoidal or spherical cells, parietal chloroplast, and reproduction by autospores [1]. It was first described in 1906 by G.A. Nadson [2]. Historically, numerous *Chloroidium* species were misclassified within other genera such as *Chlorella*, *Chlorocloster*, and *Chlorothecium* [3]. Darienko et al. (2010) [4] performed a taxonomic revision of this genus, combining morphological and ecophysiological analyses with reproductive studies and molecular characterization using 18S rRNA and ITS2 sequence data. This revision found that the genus *Chloroidium* includes four ellipsoidal *Chlorella*-like species of green microalgae. Subsequent molecular genetic studies [1] demonstrated that four additional species—the spherical *Parachloroidium laureanum* and *P. lobatum* [5], along with the ellipsoidal *Chlorella viscosum* and *C. lichinum* [6]—should be taxonomically reclassified within *Chloroidium*. In addition, a number of new species have been discovered over time: *C. antarcticum* and *C. arboricola* [1], *C. orientale* [7], *C. psoromicola* [8], *C. dehydrum* and *C. satellitum* [9]. Thus, there are currently 13 recognized species in the genus *Chloroidium*.

Members of the genus *Chloroidium* exhibit a broad geographical distribution across the Northern Hemisphere, particularly in Asia and Europe, where they colonize diverse habitats including freshwater reservoirs, soils, and biofilms on both natural (e.g., tree bark) and artificial substrates (e.g., roof tiles) [1,4,5,8,9]. Several species have been identified as lichen photobionts [1,10]. Despite this wide distribution, the genus’ biodiversity in many regions, particularly extreme environments, remains poorly characterized.

As research into the biodiversity assessment accelerates—particularly for diverse organism groups like algae –there is a growing trend toward adopting advanced methods to accurately determine taxonomic status. While traditional polyphasic taxonomic approach remains valuable, its labor-intensive nature creates a pressing need for rapid identification methods for novel taxa. This need has catalyzed the emergence of cybertaxonomy, a transformative paradigm integrating digital tools, molecular data, and machine learning to revolutionize species discovery and classification [11]. Computational species delimitation algorithms offer a powerful solution for determining species boundaries, with a multitude of methods now available for diverse organisms [12,13,14]. However, critical gaps persist in our understanding of algorithm performance across different organismal groups and phylogenetic markers. Systematic evaluation and comparative analysis of these methods’ efficacy, limitations, and optimal applications therefore represent an urgent research priority [13,14,15].

This study describes a new species of *Chloroidium* from the ecosystems of the Far North and evaluates the most effective species delimitation algorithms for this genus using standard DNA barcodes.

## 2. Results

### 2.1. Light Microscopy

**Figure 1:**
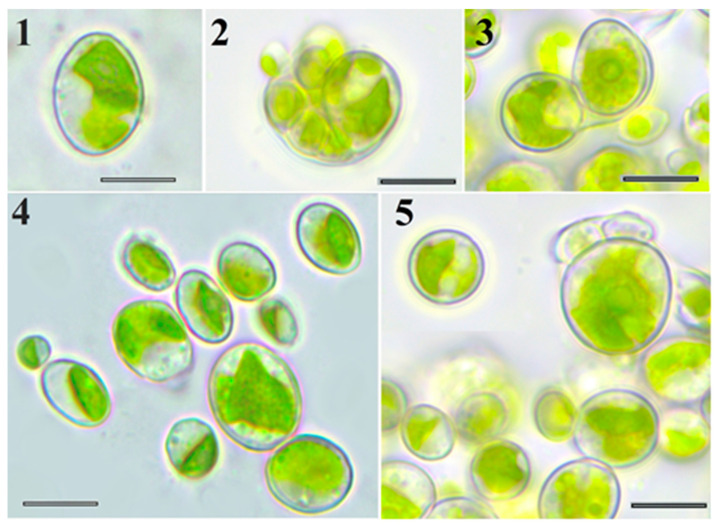
LM micrographs. Morphology of strain VKM Al-418 cells: 1—adult cell; 2,3—autosporangium; 4—21-day-old culture; and 5—6-month-old culture. Scale bar: 10 μm.

Morphological analysis identified strain VKM Al-418 as a typical ellipsoidal *Chlorella*-like microalga (Figure 1, Table 1). Young cells were ellipsoidal or broadly ellipsoidal, 4.5–9 × 2.1–8 µm. Chloroplasts of young cells were parietal, band-shaped to plate-shaped, containing a single pyrenoid with numerous starch granules. Adult vegetative cells were ellipsoidal, broadly ellipsoidal, or sometimes spherical, 9–16 × 5.1–16 µm. Chloroplasts of adult cells were parietal, band-, plate-, and lobed-shaped, with a single pyrenoid enveloped by numerous starch granules. The sporangia were broadly ellipsoidal or globose (8–18 × 6–16 μm). Asexual reproduction occurred via production of 2–16 unequal autospores (3–9 × 2–6 µm), released through sporangial wall rupture. Zoospores and sexual reproduction were not observed.

### 2.2. Morphological Clustering and Phylogenetic Incongruence

The Mantel test identified the shape and maximum size of young cells, along with the shape of adult cells, as the most significant morphological characteristics for distinguishing *Chloroidium* species (Table 2). All other measured characteristics were also statistically significant (*p* < 0.01).

**Figure 2:**
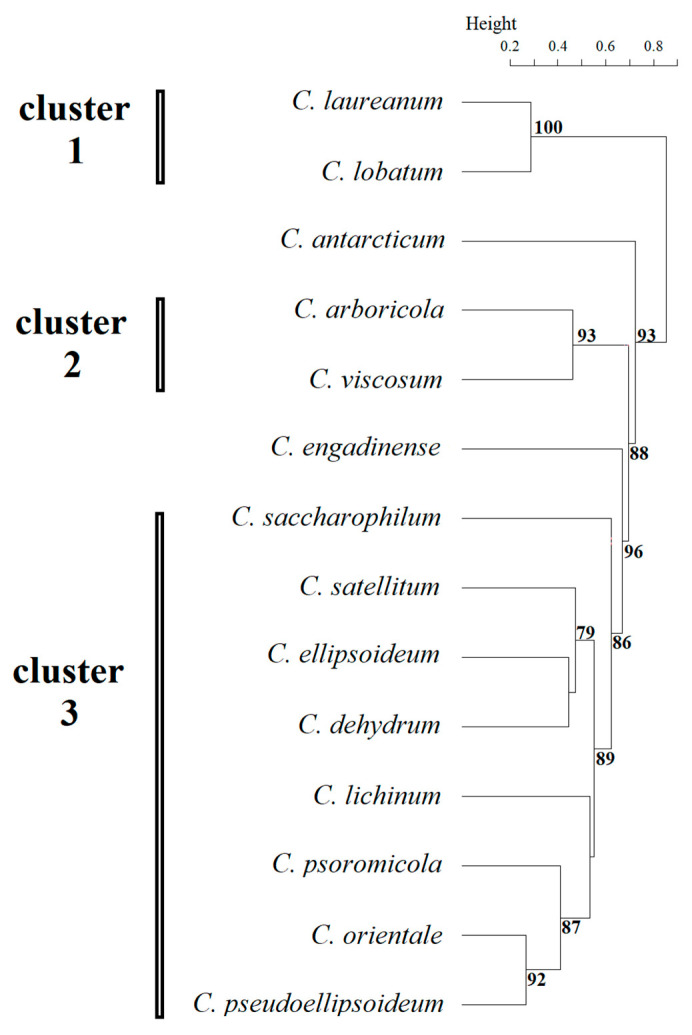
Results of clustering by morphological features of *Chloroidium* species. Statistical support values of less than 70% are not shown.

Based on these morphological data, cluster analysis revealed three well-supported main clusters and two independent lineages among the studied *Chloroidium* representatives (Figure 2).

**Figure 3:**
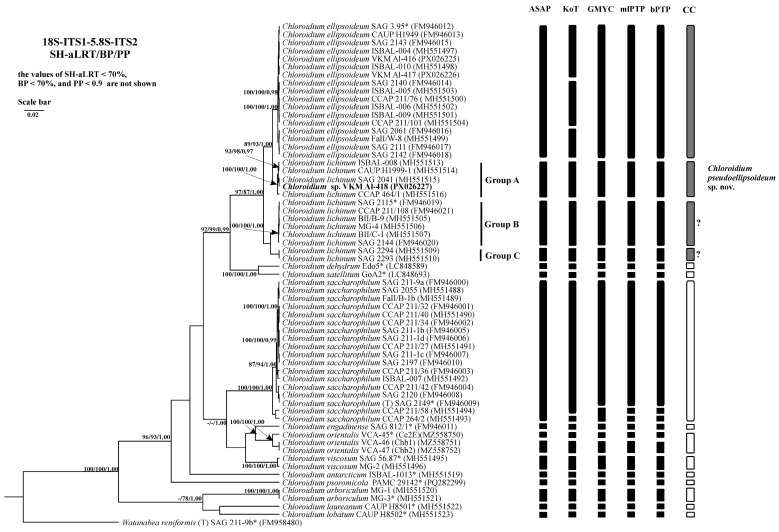
Bayesian rooted phylogenetic tree of the genus *Chloroidium* based on the 18S-ITS1-5.8S-ITS2 sequences (2729 bp). As statistical support for the nodes of the tree, SH-aLRT support (SH-aLRT), bootstrap probabilities (BPs), and posterior probabilities (PPs), respectively, are indicated; the values of SH-aLRT < 70%, BP < 70%, and PP < 0.9 are not shown. The model of nucleotide substitutions: TIM2+F+I+G4. Note: studied strain is highlighted in bold; *—authentic strains; (T)—type species. The black rectangles indicate clustering by various methods of species delimitation: ASAP, KoT, GMYC, mlPTP, and bPTP. The white rectangles show the boundaries of taxonomically recognized species that have been used to evaluate the effectiveness of delimitation algorithms. The gray rectangles and “?” indicate the species that were excluded from the analysis when evaluating the effectiveness of the delimitation algorithms. The question mark shows that the cluster needs additional study to establish clear types of boundaries.

Cluster 1 included species *C. laureanum* and *C. lobatum*, in which adult cells were spherical, the chloroplast was cup- and lobed-shaped, the pyrenoid was absent, and the number of autospores did not exceed eight. Morphological features of young cells in this cluster were not described by Neustupa et al. (2013) [5], highlighting the need for further study. Cluster 2 includes species *C. arboricola* and *C. viscosum* whose representatives had young broadly oval cells, the chloroplast of young and adult cells was band-shaped, adult cells were oval or spherical and no more than 10 microns, and the pyrenoid was naked. Cluster 3 was the most numerous, comprising seven species: *C. lichinum, C. psoromicola, C. orientale, C. saccharophilum, C. ellipsoideum, C. dehydrum,* and *C. satellitum*. The studied strain VKM Al-418, which formed an independent lineage sister to *C. orientale*, was also included in this cluster. All representatives of this cluster had young ellipsoid cells up to 11 μm in size with a band-shaped chloroplast. Spherical adult cells were also found in all representatives of the cluster, the maximum size of which ranged from 15 μm to 20 μm. The number of autospores in most representatives reached 16, excluding *C. ellipsoideum* and *C. satellitum*. *C. antarcticum* formed an independent lineage, characterized by the largest size of young and adult cells within the genus and the number of autospores (up to 64). Another independent lineage was *C. engadinense*, a distinctive feature of which was the combination of the presence of young cylindrical cells and the absence of a pyrenoid. A clear discrepancy was observed between the morphological clustering and the phylogenetic analysis (Figure 3). Tests for congruence between morphological and phylogenetic distance matrices revealed no significant correlation. Both the CADM (W = 0.407, *p* = 0.871) and Procrustes (t_0_ = 0.647, *p* = 0.594) analyses returned non-significant results, which remained non-significant after applying the Holm correction for multiple comparisons (adjusted *p* = 1.00 for both tests).

### 2.3. Phylogenetic Analysis

Phylogenetic analysis of the 18S-ITS1-5.8S-ITS2 fragment confirmed the strain’s placement within the genus *Chloroidium* (Figure 3). Strain VKM Al-418 formed a well-supported cluster (Clade A *sensu* Darienko et al. (2018) [1] sister to *C. ellipsoideum*. The genetic distances between our strain and the closely related strains were 0–0.1%, between this cluster and members of *C. ellipsoideum*—0.7–0.8%, between this cluster and members of true *C. lichinum* (Clade B, represented by a cluster with high statistical support, which includes the authentic strain of *C. lichinum* SAG 2115 and its sister strains)—0.8–0.9%, and between *C. ellipsoideum* and true *C. lichinum* (Group B)—1.1–1.2% (Appendix A Table A1).

The 18S rRNA gene of studied strain VKM Al-418 lacked introns, while intron distribution among related strains varied as follows: sister strains ISBAL-008 and SAG 2041 similarly showed no introns, whereas CAUP H1999-1 contained two introns (339 nt and 381 nt) and CCAP 464/1 possessed a single intron (381 nt) (Appendix B Table A2).

### 2.4. ITS1 Secondary Structure

**Figure 4:**
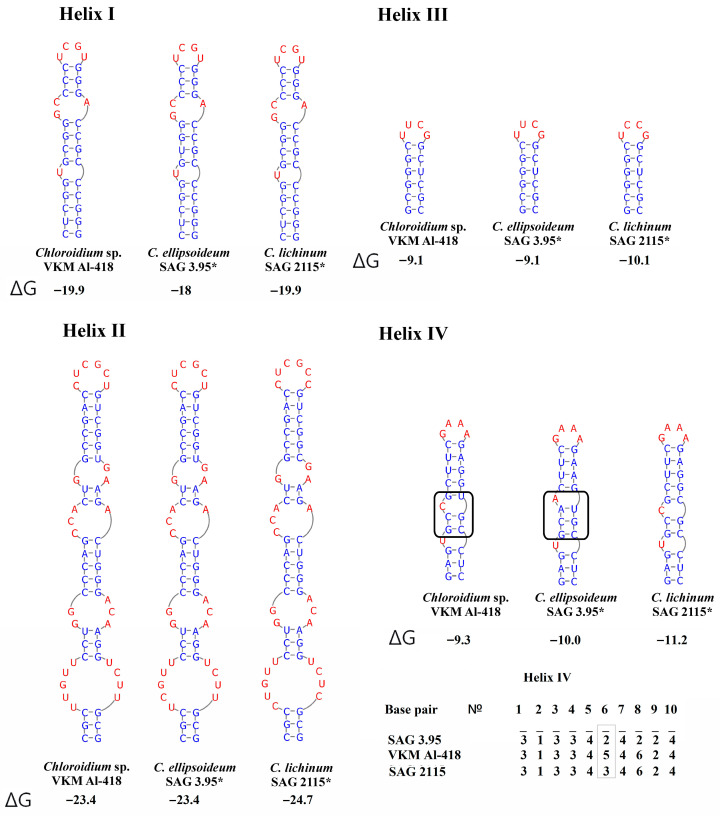
ITS1 secondary structures of *Chloroidium pseudoellipsoideum* sp. nov. VKM Al-418 and the closely related species. Note: The black rectangle indicates the differences in the secondary structure of ITS1. ΔG—a minimum free energy. Base pair—the number of nucleotide pairs in the alignment is calculated from the base of the helix. According to Darienko et al. (2018) [1], 1—A–U, 2—U–A, 3—G–C, 4—C–G, 5—G–U, and 6—U–G. Gray rectangle—a difference in all three species. *—authentic strain.

Comparative analysis of ITS1 sequences revealed a conserved lengths of 244 nt in the strain VKM Al-418, its sister strains, and in *C. ellipsoideum.* In contrast, strains of true *C. lichinum* exhibited a slightly reduced length of 243 nt. A defining characteristic of this cluster is a significantly shorter helix III compared to other *Chloroidium* species. No CBCs were detected between the VKM Al-418 cluster and either *C. ellipsoideum* or true *C. lichinum*, though structural divergence was observed in helix IV. One CBC distinguished *C. ellipsoideum* from true *C. lichinum* in this helix (Figure 4). The genetic distances between our strain VKM Al-418 and the sister strains were 2.5%, distances between this cluster and strains of *C. ellipsoideum* were 4.2–6.9%, distances between this cluster and true *C. lichinum* (clade B)—7.4–8.4%, and distances between the latter two species—8.4–9.8% (Appendix C Table A3).

### 2.5. ITS2 Secondary Structure

**Figure 5:**
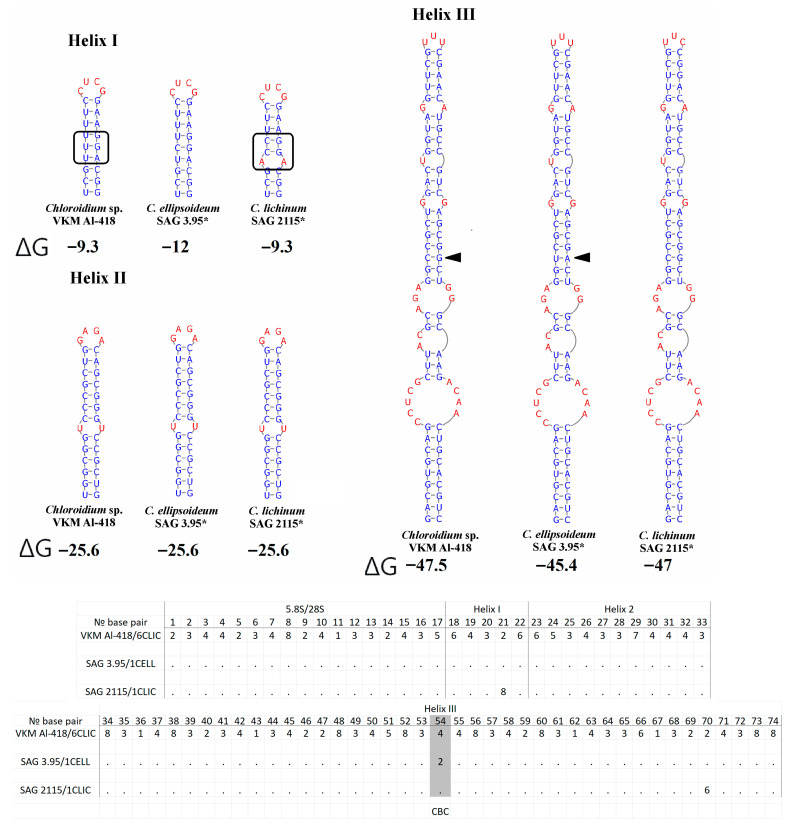
ITS2 secondary structures of *Chloroidium pseudoellipsoideum* sp. nov. VKM Al-418 and the closely related species. Note: The black arrow shows the CBC. The black rectangle indicates the differences in the secondary structure of ITS2. *—authentic strain. ΔG—a minimum free energy. № base pair—the pair number in the alignment for the entire *Choroidium* genus, based on Darienko et al. (2018) [1] and Motomatsu et al. (2025) [9]. The barcode name, according to Darienko et al. (2018) [1], is indicated by a slash from the strain number. 1—A–U, 2—U–A, 3—G–C, 4—C–G, 5—G–U, 6—U–G, 7—mismatch, and 8—deletion, single, or unpaired bases.

The ITS2 region exhibited length polymorphism: 190 nt for VKM Al-418 and sister strains, matching *C. ellipsoideum*, while true *C. lichinum* (Clade B) showed an extended 197 nt sequence. A key diagnostic feature of this strain group was the absence of helix IV, which is present in all other *Chloroidium* species. Secondary structures were conserved within the VKM Al-418 cluster, with one CBC in helix III distinguishing it from *C. ellipsoideum* (Figure 5). The genetic distances between the studied strain VKM Al-418 and the sister strains of clade A were 0–0.2%, distances between clade A and *C. ellipsoideum* were 1.9–2.4%, distances between clade A and true *C. lichinum* in clade B—3.4%, and distances between *C. ellipsoideum* and true *C. lichinum*—4.4–4.9% (Appendix D Table A4).

### 2.6. Analysis of Various DNA Barcodes to Distinguish Species

**Figure 6:**
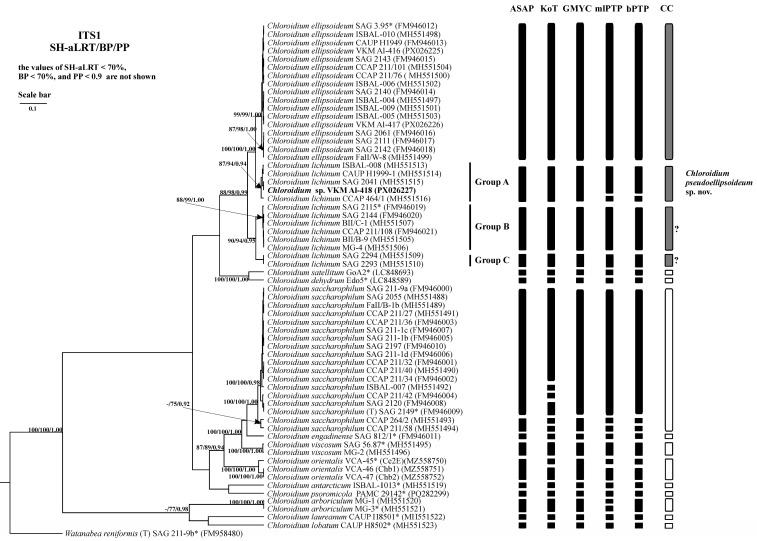
Bayesian rooted phylogenetic tree of the genus *Chloroidium* based on the ITS1 sequences (447 bp). As statistical support for the nodes of the tree, SH-aLRT support (SH-aLRT), bootstrap probabilities (BPs), and posterior probabilities (PPs), respectively, are indicated; the values of SH-aLRT < 70%, BP < 70%, and PP < 0.9 are not shown. The model of nucleotide substitutions: TIM2+F+G4. The black rectangles indicate clustering by various methods of species delimitation: ASAP, KoT, GMYC, mlPTP, and bPTP. The white rectangles show the boundaries of taxonomically recognized species that have been used to evaluate the effectiveness of delimitation algorithms. The gray rectangles and “?” indicate the species that were excluded from the analysis when evaluating the effectiveness of the delimitation algorithms. The question mark shows that the cluster needs additional study to establish clear types of boundaries. *—authentic strains.

**Figure 7:**
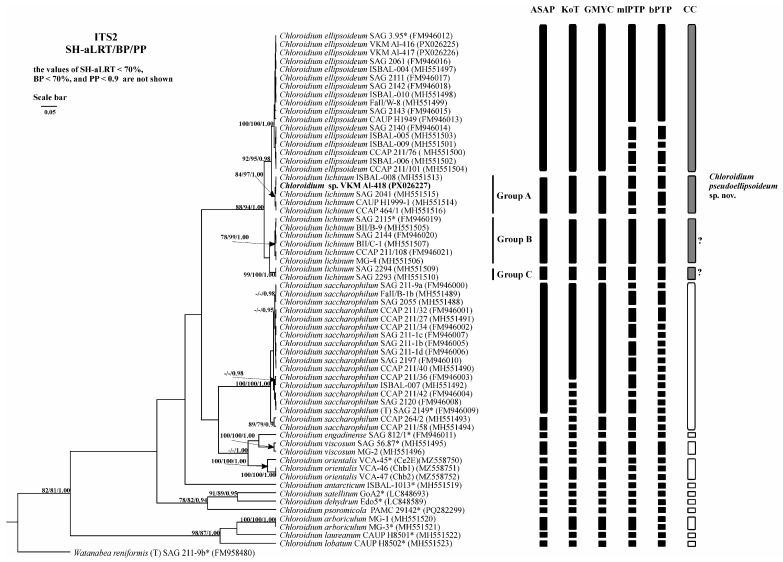
Bayesian rooted phylogenetic tree of the genus *Chloroidium* based on the ITS2 sequences (335 bp). As statistical support for the nodes of the tree, SH-aLRT support (SH-aLRT), bootstrap probabilities (BPs), and posterior probabilities (PPs), respectively, are indicated; the values of SH-aLRT < 70%, BP < 70%, and PP < 0.9 are not shown. The model of nucleotide substitutions: TIM2+I+G4. The black rectangles indicate clustering by various methods of species delimitation: ASAP, KoT, GMYC, mlPTP, and bPTP. The white rectangles show the boundaries of taxonomically recognized species that have been used to evaluate the effectiveness of delimitation algorithms. The gray rectangles and “?” indicate the species that were excluded from the analysis when evaluating the effectiveness of the delimitation algorithms. The question mark shows that the cluster needs additional study to establish clear types of boundaries. *—authentic strains.

**Figure 8:**
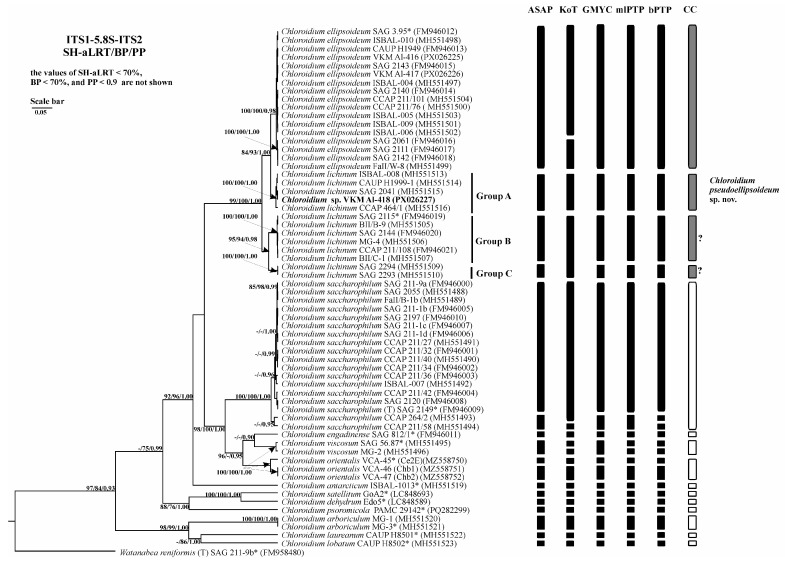
Bayesian rooted phylogenetic tree of the genus *Chloroidium* based on the ITS1-5.8S-ITS2 sequences (944 bp). As statistical support for the nodes of the tree, SH-aLRT support (SH-aLRT), bootstrap probabilities (BPs), and posterior probabilities (PPs), respectively, are indicated; the values of SH-aLRT < 70%, BP < 70%, and PP < 0.9 are not shown. The model of nucleotide substitutions: TIM2+F+I+G4. The black rectangles indicate clustering by various methods of species delimitation: ASAP, KoT, GMYC, mlPTP, and bPTP. The white rectangles show the boundaries of taxonomically recognized species that have been used to evaluate the effectiveness of delimitation algorithms. The gray rectangles and “?” indicate the species that were excluded from the analysis when evaluating the effectiveness of the delimitation algorithms. The question mark shows that the cluster needs additional study to establish clear types of boundaries. *—authentic strains.

Comparative evaluation of five barcode regions revealed overlapping intra- and interspecific genetic distances across all markers (Table 3). The regions V4 and V9 of the 18S rRNA gene showed high conservation, with extensive distance overlap and poor discriminatory power for *Chloroidium* species. In contrast, ITS1, ITS2, and the concatenated ITS1-5.8S-ITS2 region exhibited substantially higher variability, enabling species-level identification with maximal efficiency (Figure 6, Figure 7 and Figure 8). Among these three variable markers, ITS1 and ITS1-5.8S-ITS2 demonstrated the smallest distance overlap, while the ITS2 region showed marginally greater overlap (Table A3, Table A4 and Table A5).

### 2.7. Species Delimitation

The threshold-based ASAP algorithm demonstrated the highest congruence with current *Chloroidium* taxonomy for concatenated 18S-ITS1-5.8S-ITS2 fragment (90.9% match rate) (Table 4). When analyzing the fragment ITS1-5.8S-ITS2 and the spacer ITS1, ASAP (81.8% and 90.9%, respectively) and GMYC (81.8% and 90.9%, respectively) were the most accurate. When using the ITS2 spacer as a phylogenetic marker, the efficiency of the algorithms was lower and did not exceed 81.8%. The efficiency of the three algorithms was the same. At the same time, the results of the ASAP and GMYC methods were the same, and the mlPTP algorithm tended to divide the species into more MOTUs. Therefore, in this case, despite the same efficiency, the use of the first two algorithms is preferable.

### 2.8. Habitat

The VKM Al-418 strain was isolated from cryogenic crack soil of Yakutia. At the time of sampling, the habitat conditions in this biotope could be characterized as humid (up to 100%) and relatively cool (7.2–11.1 °C). At the same time, the soil pH did not exceed 4. The remaining closely related strains were isolated from warmer climates. The sister strains SAG 2041, CAUP H1999-1, and CCAP 464/1 were isolated from the temperate zone, while the strain ISBAL-008 was found in the tropical zone. The habitat of the sister strains varied. The strains SAG 2041, ISBAL-008, and CAUP H1999-1 were epiphytes on various substrates (roof, volcanic ash, and tree bark, respectively). At the same time, the strain CCAP 464/1 was an inhabitant of a freshwater reservoir.

### 2.9. Fatty Acid Composition

The FA composition of the strain VKM Al-418 grown under different temperature regimes (9 °C, 22 °C, and 27 °C) is presented in Table 5. The total fatty acid content was 6.79 ± 0.61% of the biomass dry weight at 9 °C and 5.48 ± 0.08% at 22 °C. Growth at 27 °C was very poor, yielding insufficient biomass for gravimetric analysis to determine the total FA content (as a percentage of cell dry weight); therefore, only the relative FA composition was determined for this temperature. Palmitic, oleic, and linoleic (LA) acids were the main FAs (≥5% of total FAs) under all studied conditions. Cells grown at 9 °C exhibited a fatty acid profile dominated by α-linolenic acid (ALA; 41.6%, CI 36.6–46.6%), which was significantly higher than in other variants (*p* < 0.01). The other major FAs were palmitic acid (20.6%, CI 16.9–25.3%) and LA (17.1%, CI 14.9–19.3%). This variant was also characterized by the highest level of 7,10,13-hexadecatrienoic acid (HDTA; 6.0%, CI 3.8–8.2%, *p* < 0.01) and a low content of oleic acid (5.7%, CI 2.5–8.9%). Consequently, it exhibited the highest unsaturation index (UI; 1.892, CI 1.706–2.078, *p* < 0.05).

Compared to the 9 °C treatment, cells grown at 22 °C exhibited a significantly lower ALA content (22.8%, CI 19.3–26.3%, *p* < 0.01) and a higher LA content (36.1%, CI 30.6–41.6%, *p* < 0.01). The HDTA content also decreased notably to 1.7% (CI 0.7–2.7, *p* < 0.05). The UI for this variant was 1.623 (CI 1.459–1.787), a small yet statistically significant decrease from the value at 9 °C (*p* < 0.05). Cells grown at 27 °C were distinguished by the highest content of palmitic (41.8%, CI 33.1–50.5%, *p* < 0.01) and oleic (25.4%, CI 17.9–32.9%, *p* < 0.01) acids among all treatments. Conversely, the content of trienoic FAs dropped significantly: HDTA was nearly undetectable (CI −0.1–0.3%, *p* < 0.05), and ALA decreased to 3% (CI 0.3–5.7, *p* < 0.01). In contrast, the LA content (21.2% CI 11.3–31.1) was not significantly different from the 9 °C variant. As a result of these FA composition shifts, cells cultured at 27 °C exhibited the lowest UI (0.802, CI 0.566–1.038, *p* < 0.01).

## 3. Discussion

The morphology of the studied strain VKM Al-418 conformed to that of typical representatives of the genus *Chloroidium*. As previously reported (Table 1), species within this genus can be categorized by the presence or absence of a pyrenoid and whether the pyrenoid is enveloped by starch grains. The strain VKM Al-418 belonged to the group characterized by the presence of a pyrenoid surrounded by starch grains. This strain differed from the closely related species *C. lichinum* and *C. ellipsoideum*, particularly in the shape and size of young and adult cells. Unlike these species, VKM Al-418 lacked ovoid and irregular cells. Its cells were slightly larger than those of *C. ellipsoideum* but smaller than those of *C. lichinum*, and it produced a larger number of autospores than *C. ellipsoideum*. According to the Mantel test, the most discriminative morphological characteristics were the shape and maximum size of young cells and the shape of adult cells. Consistent with patterns observed in other microalgae, the clustering based on these morphological features showed incongruence with the phylogenetic topology, a phenomenon previously documented in the genus *Coelastrella* (Chlorophyceae, Chlorophyta) [16,17]. Despite this overall discordance, the morphological analysis supported the distinctness of VKM Al-418, as it consistently formed a distinct lineage sister to *C. orientale* (Figure 2). The problem of phenotypic polymorphism in representatives of the genus *Chloroidium* and, as a consequence, the lack of clear morphological characteristics for the unambiguous delineation and identification of species was previously raised by Darienko et al. (2018) [1]. Such polymorphism may be an adaptive response to climatic factors during range expansion. Due to the difficulty of identifying species based on morphological characteristics, a recommendation was made to pay particular attention to molecular data [1].

Results of phylogenetic analyses, based on the concatenated 18S-ITS1-5.8S-ITS2 fragment, are consistent with the findings of Darienko et al. (2018) [1], Gontcharov et al. (2021) [7], and Chae et al. (2025) [8]. In all these studies, the cluster containing the studied strain VKM Al-418 (designated as Group A [1]) consistently formed a well-supported, closely related lineage with *C. ellipsoideum*, but never grouped with *C. lichinum*. The previous assignment of this cluster to *C. lichinum* was based solely on a shared ITS2 molecular signature and was not supported by its phylogenetic position within the genus *Chloroidium* in any of the referenced studies. Instead, the positioning of this cluster, which includes strain VKM Al-418, indicates that its members constitute a distinct species, sister to both *C. ellipsoideum* and *C. lichinum*. Notably, strains SAG 2294 and SAG 2293, classified under Group C [1], consistently lacked strong statistical support in all analyses [1,7,8] despite clustering with confirmed *C. lichinum* representatives (Group B). This discrepancy may indicate the existence of an additional cryptic species requiring further investigation.

Although introns have been used as an additional tool for species identification in some green algal groups [16,17,18,19,20], this approach is not applicable to members of the genus *Chloroidium*. For instance, *C. saccharophilum* exhibits considerable intron variability, with some strains lacking introns entirely, others possessing one, and some harboring two. Consequently, the difference in intron number between strain VKM Al-418 and its closely related strains CAUP H1999-1 and CCAP 464/1 likely reflects intraspecific variation rather than a species-level distinction.

The studied strain VKM Al-418, along with other strains in Clade A, differed from *C. ellipsoideum* not only in structural features of helix IV of ITS1, but also by possessing one CBC in the conserved helix III of ITS2. Given that even consistent spacer structure variations can suggest species-level divergence, the presence of a CBC in the conserved ITS2 region provides particularly strong evidence for distinct species status [19,20,21,22]. Within the genus *Chloroidium*, CBCs in ITS1 were infrequent, occurring only between a few species. For instance, no CBCs were detected between closely related species such as *C. antarcticum* and *C. ellipsoideum*, *C. psoromicola* and *C. engadinense*, or *C. lobatum* and *C. orientale*. In contrast, CBCs were identified between certain strains within the species *C. orientale* and *C. saccharophilum*. Given the high intraspecific distances observed in these species, this finding suggests they may contain cryptic diversity warranting further investigation. In the ITS2 region, the presence of at least one CBC between different species was common, though not universal; for example, no CBCs were found between *C. arboricolum* and *C. laureanum*. In conclusion, while the presence of CBCs and other stable differences in the secondary structure of internal spacers are valuable taxonomic indicators, they are most informative when used as part of a comprehensive, integrative approach to corroborate the independent status of a new species.

To date, research on green algae has primarily focused on species that underwent long-term independent evolution, while evolutionarily young species remain understudied [12,13,14,16]. This bias has complicated the establishment of reliable interspecific distance thresholds for both the extended 18S-ITS1-5.8S-ITS2 fragment and for shorter barcodes. Within the *C. ellipsoideum*/*C. lichinum* complex (encompassing Groups A, B, and C), we identified minimum interspecific distances for each of these regions. Importantly, phylogenetic tree topology and ITS1/ITS2 secondary structure analyses unambiguously support the recognition of Groups A and B of *C. lichinum* as distinct species. The taxonomic status of Group C requires further study. The observed genetic distances between *C. ellipsoideum* and Group A are minimal within the genus: for 18S-ITS1-5.8S-ITS2 ≥0.7%, for ITS1 ≥4.2%, for ITS2 ≥ 1.9%, and for ITS1-5.8S-ITS2 ≥ 2.3%. The optimal ROC thresholds for all markers (0.65% for the 18S-ITS1-5.8S-ITS2 fragment, 4.6% for ITS1, 3.8% for ITS2, and 3.0% for the ITS1-5.8S-ITS2 fragment) were close to these values (Appendix F Table A6). Recognizing Group A as a separate species provides a clearer framework for delineating species within the genus. Under the previously accepted concept of the *C. lichinum* species, the distances within the cluster, including Groups A, B, and C, exceeded the distances between this cluster and *C. ellipsoideum*. At the same time, within the genus at this stage of study, the vast majority of species are characterized by a high level of interspecific genetic differences. For example, *C. viscosum*, which in the sequence of genetic distances comes right after the studied cluster, demonstrates a significantly higher level of genetic differences: 1.8% for the 18S-ITS1-5.8S-ITS2 fragment, 9.1% for ITS1, 15.8% for ITS2, and 9.4% for the ITS1-5.8S-ITS2 fragment. It can also be assumed that, as data accumulates, the range of intraspecific variability will increase. Therefore, it is not possible to focus only on genetic distances to describe new taxa. Instead, the status of a new taxon should be confirmed through a polyphasic approach and based on a combination of evidence.

Analysis of commonly used DNA barcodes has shown that the internal transcribed spacers ITS1, ITS2, and the concatenated fragment ITS1-5.8S-ITS2 are optimal for distinguishing species within the genus *Chloroidium*, offering several advantages over alternative markers: (1) their relatively short length; (2) high variability; (3) substantial database representation; and (4) minimal overlap between intra- and interspecific genetic distances according to updated thresholds. Among them, the use of the concatenated ITS1-5.8S-ITS2 fragment is particularly valuable because it allows us to analyze the features of the secondary structures of internal transcribed spacers, including ITS2, for which a generally accepted model of the secondary structure has been developed. In contrast, the V4 and V9 regions of 18S rRNA are not suitable as barcodes for this genus owing to their extreme conservation. The V4 region’s utility is further compromised by frequent intron insertions in multiple species (for example, *C. saccharophilum*, *C. laureatum*, and *C. ellipsoideum*), while the V9 region’s terminal position in the 18S rRNA gene presents technical challenges, including incomplete sequencing in some strains (particularly *C. saccharophilum*) and intron presence in others.

Among the mathematical algorithms evaluated for species delimitation, the ASAP algorithm demonstrated superior performance for both long (18S-ITS1-5.8S-ITS2) and short (ITS1, ITS2, ITS1-5.8S-ITS2) barcodes. The GMYC algorithm also showed good efficiency for the shorter barcodes, but its implementation is more labor-intensive, requiring the preliminary construction of ultrametric trees. In contrast, ASAP operates directly on genetic distance matrices via a user-friendly online platform. Therefore, we recommend using ASAP as the primary species delimitation tool for the *Chloroidium* genus, particularly during the initial stages of dataset assembly and analysis. Once a final dataset is established, GMYC can serve as a valuable supplementary method. Furthermore, given that GMYC has been shown to be more accurate at delineating evolutionarily young and morphologically similar species [16,23], its usefulness for the genus *Chloroidium* is likely to increase as more diversity is sampled and such complexes are identified. It is important to emphasize that the outputs of these algorithms should be interpreted as hypotheses that should be integrated with morphological, ecological, and other molecular data, rather than as definitive evidence for taxonomic decisions.

Members of the genus *Chloroidium* have a broad geographic distribution across diverse habitats (soil, phytoplankton, fouling, and various substrates). The studied strain, isolated from soils in Northern Yakutia, represents a typical example of this ecological versatility. Closely related strains have been documented throughout Europe, primarily forming biofilms on building surfaces, volcanic ash, and other substrates. The species *C. ellipsoideum* exhibits a particularly wide distribution, being found across Europe, Asia, and Africa in various ecological contexts—from terrestrial biocrusts and biofilms to shallow pond phytoplankton. In contrast, *C. lichinum* has a more restricted distribution in Europe, mainly occurring as a component of fouling on tree bark, with occasional occurrences on rocks and other substrates [1,4].

Because the studied strain originates from the Far North and FA composition is crucial for low-temperature adaptation of photosynthetic organisms [24,25,26], we examined its FA profile under different temperature regimes of cultivation (9 °C, 22 °C, and 27 °C). Strain VKM Al-418 exhibited an FA profile typical of green algae, dominated by palmitic, oleic, linoleic, and α-linolenic acids. A similar set of major FAs was found in all studied *Chloroidium* strains: *C. saccharaphilum* SAG 211-1d, SAG 2055, and SAG 2120 [27]; Coliumo strain [28]; MACC 477 [26]; *C. lichinum* SAG 2041 [27]; *C. engadiensis* SAG 812-1 [27]; *C. viscosum* SAG 2338 [29]; and *Chloroidium* sp. UTEX 3007 [30]. In contrast, oleic acid is present in low amounts (<5%) or is absent in strains of *C. saccharaphilum* SAG 211-1b, SAG 211-1c, and SAG 211-9a; *C. ellipsoideum* SAG 3.95; and *C. viscosum* SAG 56.87 [27]. The reported absence of oleic acids is likely erroneous, as it is a necessary precursor for the biosynthesis of linoleic acid and ALA [31]. The presence of 16-carbon polyunsaturated FAs (16:2 and 16:3) in *Chloroidium* representatives appears to be rare, having been reported only by Lang et al. (2011) [27] and in this study. The absence of these FAs in other studies is likely attributable to their low abundance and potential misidentification in chromatograms due to co-elution with predominant FAs or a lack of appropriate standards. Notably, temperature exerted a considerable impact on the FA profile of the studied strain, underscoring its adaptive response to a harsh environment.

At low temperature (9 °C), the strain exhibited maximal levels of trienoic FAs (HDTA and ALA) and the highest unsaturation index. This pattern indicates an adaptation to cold, which serves to maintain photosynthetic membrane fluidity, as polyunsaturated HDTA and ALA increase in membrane fluidity and typically esterify plastidic galactolipids [32]. Notably, the ALA content in strain VKM Al-418 grown at 9 °C was the highest among all reported *Chloroidium* FA profiles [26,27,28,29,30]. Under warmer conditions (22 °C), the content of both trienoic FAs dropped significantly, while the LA content increased. This suggests that ω-3 desaturase, the enzyme responsible for introducing the third double bond to convert LA into ALA, is temperature-dependent. It is worth noting that many reported *Chloroidium* strains are characterized by a high content of linoleic acid [26,27,28]. The higher temperature of 27 °C was stressful for strain VKM Al-418, as evidenced by its ceased growth. This stress was further reflected in the FA profile through the accumulation of high amounts of palmitic and oleic acids and a dramatic decrease in ALA content. This pattern indicates the inhibition of both elongation and desaturation reactions at 27 °C. A similar trend in the changes of palmitic, linoleic and α-linoleic acids was observed in *Chlorella* sp. MACC 800 cultivated at 15 °C, 25 °C, and 35 °C [26], where the palmitic acid content reached 40.7% at 35 °C. Among *Chloroidium* strains, a similarly high palmitic acid content (41.8%) has been reported only in *Chloroidium* sp. UTEX 3007, a strain isolated from a desert region in the United Arab Emirates [30]. The authors suggested that such a high palmitic acid content enables survival in a desert climate, as this FA is more thermostable and provides necessary membrane stability under high-temperature conditions. It is noteworthy that this high palmitic acid content was observed at 25 °C and 400 μmol photons m^−2^ s^−1^, conditions which are not extreme for a desert strain. Therefore, the question of whether palmitic acid accumulation in VKM Al-418 at 27 °C is a part of a specific adaptive mechanism to high temperature or merely a consequence of stress-induced inhibition of elongation and desaturation enzymes requires further investigation.

## 4. Materials and Methods

### 4.1. Isolation and Cultivation of Algal Strains

The studied algal strain was isolated from soil in 2020 from peat cryozems (yedoma) near the lower reaches of the Kolyma River, Duvannyi Yar, Sakha Republic, Russia (68°37′51″ N 159°5′4″ E). The study area belongs to the northern taiga zone and has a continental climate. According to the Cherskiy Weather Station, the average annual air temperature is −10.3 °C and the average annual precipitation is 236 mm. The layer of active seasonal melting ranges from 30 cm to 50 cm in thickness. The study site is located on a slightly convex slope that is covered by sparse larch trees with a predominance of dead wood and partially moss and lichen ground cover. Samples of soil, including peat and litter, were taken from the upper 6 cm layer of peat cryozem located above a 30 cm deep interpolygonal cryogenic crack. The material was collected during the period of maximal seasonal thawing (in September 2019). The samples were placed in sealed sterile bags, stored and transported at low positive temperatures (2–4 °C) and humidity in the field. A small amount of the soil sample was re-wetted with sterile MilliQ water and plated on Petri dishes with solid BG-11 medium (1% agar) [33]. Unialgal culture was established through serial transfer and colony isolation, followed by incubation at 23–25 °C under a 12:12 h light/dark photoperiod (60–75 μmol photons m^−2^ s^−1^, cool white fluorescent illumination). The isolated strain was deposited in the All-Russian Collection of Microorganisms (VKM) at the Skryabin Institute of Biochemistry and Physiology of Microorganisms (Pushchino, Russia), under accession number VKM Al-418.

### 4.2. Light Microscopy (LM)

Morphological characterization and life cycle analysis were conducted using a Leica DM750 light microscope (Wetzlar, Germany) equipped with a Leica Flexacam C3 color digital camera (Wetzlar, Germany). The observations of the strain were performed from two weeks to six months post-inoculation. Two hundred cells of the strain were measured for size comparison.

### 4.3. Statistical Analysis of Morphological Traits and Their Congruence with Phylogeny

For comparative analysis, the characteristics of the strains were encoded in the form of binary vectors. The length of the binary vector of the analyzed feature was equal to the number of its possible states, while each element corresponded to a certain state. For the analyzed strains, 1 was recorded in the position corresponding to the characteristic’s state, while the remaining elements had a value of 0. All binary vectors determining the states of each trait for all strains studied in the analysis were summarized in a single table. The analysis used strains for which the states of 80% or more of the considered traits were known, the remaining strains were excluded from the analysis. On the basis of the binary table of feature states, the similarity and difference in strains were visualized using multidimensional scaling, for which a matrix of Jaccard distances was used (one minus the share of common non-zero states in the total number of non-zero states in the two strains being compared), excluding indeterminate features for each pair of strains. In order to determine the significance of a trait in the overall distribution of distances between strains, the Mantel test [34,35] based on the Pearson correlation coefficient was used. The reliability of the correlation was assessed using a permutation test with 10,000 replicates. During the Mantel test, the general Jaccard distances matrix was compared with distance matrices calculated for each feature separately. The higher the value of the Pearson correlation coefficient for the trait under consideration, the greater the contribution it makes to the separation of strains. All calculations were performed using the “vegan” package [36] in the R programming language. A dendrogram was generated using UPGMA (unweighted pair-group method with arithmetic averages) in cluster analysis [37], also implemented in R [38].

To determine the congruence between phylogenetics and morphology, we compared two distance matrices: patristic distances computed from the species phylogeny (cophenetic.phylo), R package ape ver. 4.4.1 [39]) and feature states (vegdist(), method = “jaccard”, and R package vegan [36]). Global congruence was assessed with two independent tests: CADM (CADM.global() and R package ape [39]) and Procrustes analysis (protest() and R package vegan [36]). Significance was evaluated by permutation (9999 permutations). Because we performed two global tests of the same hypothesis (CADM and Procrustes), we controlled the family-wise error rate using the Holm correction (FWER) implemented in R (p.adjust(), method = “holm”, and R package stats).

### 4.4. DNA Isolation, Amplification, Purification, and Sequencing

Total DNA was extracted using the DNeasy Plant Mini Kit (Qiagen, Germantown, MD, USA) according to the manufacturer’s protocol. Primers for amplification of the 18S rRNA gene were described by Katana et al. (2001) [40]: F 5′-GCGCTACCTGGTTGATCCTGCCAGT-3′, R 5′-GATCCTTCTGCAGGTTCACCTAC-3′; amplification conditions: 95 °C, 5 min; 95 °C (30 s), 55 °C (30 s), 72 °C (2 min), 35 cycles; 72 °C, 5 min. Primers for amplification of the internal transcribed spacer 1 (ITS1), 5.8S rRNA gene, and internal transcribed spacer 2 (ITS2) were described by Johnson et al. (2007) [41]: ITS–AF 5′-CGTTTCCGTAGGTGAACCTGC-3′, ITS–BR 5′- CATATGCTTAAGTTCAGCGGG-3′; amplification conditions: 95 °C, 3 min; 95 °C (30 s), 57.6 °C (30 s), 72 °C (1 min), 35 cycles; 72 °C, 10 min. The target PCR products were electrophoretically verified on 1% agarose gel, purified using the Cleanup Mini kit (Evrogen, Moscow, Russia), and commercially sequenced (Sanger method) by Evrogen (Moscow, Russia).

### 4.5. Phylogenetic Analysis

Phylogenetic analysis was performed on concatenated 18S-ITS1-5.8S-ITS2 sequences, with the closest homologs identified via BLASTn in GenBank (Bethesda, Maryland, MD, USA) (https://blast.ncbi.nlm.nih.gov, accessed on 10 November 2025) using strict selection criteria (≥95% similarity, no ambiguous bases, minimum 2200 bp length, and preference for type species and collection authentic strains). Introns in 18S rRNA gene were excised prior to alignment, and taxonomic nomenclature followed AlgaeBase [3]. Multiple alignment was performed using MAFFT [42,43]. Tree topologies were cross-validated against published data [1,4,5,7,8,9]. Optimal model was selected based on the minimum values of the Bayesian Information Criterion (BIC). For maximum likelihood (ML) analysis, we used the IQ-TREE ver. 2.2 [44]. As a branch support test, we conducted 10,000 replicates of the UFboot2 bootstrap test [45]. Topology estimation for ML trees was based on 1000 replicates of the SH-aLRT test [46], implemented in the Building Phylogenetic Tree block of the uGENE. Bayesian inference (BI) was carried out using the BEAST2 ver. 2.7.5 software package [47]. Analysis included running the Markov chain using the Monte Carlo method (MCMC). Markov Chain Monte Carlo analyses in BEAST2 were run for 100 million generations (18S-ITS1-5.8S-ITS2 and ITS1-5.8S-ITS2), 180 million generations (ITS1), and 200 million generations (ITS2). Parameters and trees were sampled every 10,000 steps following a 25% burn-in. The effectiveness of MCMC on the convergence of the results with the estimated effective sample size (ESS) for all parameters above 200 was carried out with the Tracer ver. 1.7 program [48]. A consensus tree was computed based on the maximum confidence clade (MCC) using TreeAnnotator ver. 2.7 with 25% burn-in [49]. Genetic differences between nucleotide sequences were characterized using genetic distances (using the Kimura 2-parameter model), which were calculated in MEGA 11.0 [50].

Statistical analysis was performed to evaluate the discriminatory power of genetic markers for taxon delimitation. We used Receiver Operating Characteristic (ROC) curve analysis and Area Under the Curve (AUC) evaluation with calculation of sensitivity and specificity. Data preparation: Based on the pairwise genetic distance matrix, we created two vectors: a vector of genetic distances and a vector of binary labels, where 0 corresponded to intraspecific pairs and 1 to interspecific pairs. ROC analysis was performed using the pROC package in the R environment [51]. AUC with a 95% confidence interval was calculated using DeLong’s method [52]. The optimal genetic distance threshold was determined using Youden’s J statistic, which maximizes the sum of sensitivity and specificity. Distribution overlap analysis was conducted using the overlapping package (v1.5.0) [53], calculating the overlap area between intraspecific and interspecific genetic distance distributions. Additionally, we determined the overlap minimizing threshold as the arithmetic mean between the maximum intraspecific and minimum interspecific distance. Interpretation of results: AUC > 0.9 was considered indicative of excellent discriminatory power, AUC > 0.8 of good power, and AUC = 0.5 of no discriminatory power [54].

### 4.6. Folding of ITS1 and ITS2 Secondary Structures

Secondary structures of ITS1 and ITS2 were predicted using the RNAfold web server (http://rna.tbi.univie.ac.at//cgi-bin/RNAWebSuite/RNAfold.cgi, accessed on 20 November 2025) based on minimum free energy principles, with structural validation following Coleman (2015) [21] for ITS1 and Caisova et al. (2013) [55] for ITS2. Comparative analysis of spacer structures, including conserved motif identification and compensatory base change (CBC) detection, was carried out in 4SALE [56,57]. For ITS2-based species delimitation, we applied the Coleman (2000) [58] and Demchenko et al. (2012) [59] criteria, where a single CBC in conserved regions (5 bp of helix I, 10 bp of helix II, entire helix III) indicates sexual incompatibility. The secondary structures were visualized using PseudoViewer3, and genetic distances (using the Kimura 2-parameter model) were calculated in MEGA 11.0 [50].

### 4.7. Species Delimitation

This study employed five algorithms for species delimitation: (1) a population genetic theory approach calculating the K/θ ratio (average pairwise distance between putative species-level clades to genetic diversity estimate) performed using the package ‘KoT-K over Theta’ ver. 1 [60]; (2) the threshold-based ASAP, (Assemble Species by Automatic Partitioning) algorithm [61], implemented via the ‘asapy’ script of the iTaxoTools package ver. 0.1 [62]; (3) the Generalized Mixed Yule Coalescent (GMYC) method; and (4–5) Poisson tree processes (mlPTP and bPTP) applied to an IQ-TREE-derived ML phylogeny on an online server at https://species.h-its.org/ (accessed on 10 November 2025) [63]. All analyses followed the protocols of [16], with resulting MOTUs classified against morphological taxonomy as matches (concordant units), splits (species divided), merges (species combined), or mixtures (split/merge combinations) [64].

### 4.8. Determination of the Total Lipid (TL) Content and the Fatty Acid (FA) Composition of TL

Cells of the studied strain VKM Al-418 were harvested from the Petri dish and resuspended in 1 mL of BBM-3N medium. From this suspension, 100 μL aliquots were transferred into 50 mL of fresh BBM-3N medium in Erlenmeyer flasks, with three biological replicates prepared for each condition. The cultures were grown for 21 days in temperature-controlled systems under continuous illumination of 30 μmol photons m^−2^ s^−1^ provided by warm white LED lights. Three temperature regimes were applied: 9 °C, 22 °C, and 27 °C. After the incubation period, samples were taken to measure biomass concentration (9 °C and 22 °C) and determine FA profiles (all conditions). FAs of the cell lipids were extracted and determined by GC-MS of the FA methyl esters as described previously [16], using margaric acid as an external standard. To measure the dry weight, the samples were precipitated by centrifugation, washed with distilled water, and dried at 80 °C overnight in pre-weighed plastic microtubes in a heat oven.

Data are presented as mean ± standard deviation (SD; *n* = 3). For the main fatty acids, statistical significance was assessed by one-way ANOVA followed by Tukey’s HSD post hoc test, performed using the online tool at https://astatsa.com/OneWay_Anova_with_TukeyHSD/ (accessed on 17 November 2025). The effect size for the ANOVA was calculated as eta squared (η^2^). The 95% confidence intervals (CIs) were calculated based on the t-distribution. Note that some lower CI bounds are negative due to the sample size and data variability; these biologically implausible values indicate that the true mean is close to zero.

## 5. Conclusions

Thus, the studied strain VKM Al-418 is described as a new species, *C. pseudoellipsoideum* sp. nov. This description is supported by polyphasic approach, which combines distinct morphological characteristics, a robust phylogenetic placement based on the 18S–ITS1-5.8S-ITS2 fragment, unique features in the secondary structures of both ITS1 and ITS2—including a compensatory base change (CBC) in the highly conserved helix III of ITS2—and confirmation by five independent species delimitation algorithms.

Our analyses further revealed a significant incongruence between morphological and phylogenetic patterns, underscoring the limitations of relying solely on phenotypic data for species identification in this genus. A comparative evaluation of DNA barcodes established the internal transcribed spacers (ITS1, ITS2, and the concatenated ITS1-5.8S-ITS2 fragment) as optimal markers for species discrimination, in contrast to the highly conserved and thus less informative V4 and V9 regions of the 18S rRNA gene. Among the delimitation algorithms tested, ASAP demonstrated superior performance, offering an optimal balance of accuracy and computational efficiency for preliminary assessments. The GMYC algorithm also demonstrated high efficiency, albeit with a more computationally intensive and time-consuming implementation.

Based on our comprehensive analyses, we propose the following revised new minimum of interspecific genetic distances within the genus *Chloroidium*: 0.7% for 18S-ITS1-5.8S-ITS2 fragment, 4.2% for ITS1, 1.9% for ITS2, and 2.3% for ITS1-5.8S-ITS2 fragment. However, given the observed overlap between intra- and interspecific distances, these values should serve as guidelines rather than sole criteria for species delimitation. While mathematical algorithms like ASAP are invaluable for generating initial hypotheses, their results must be interpreted as part of a broader evidential framework. Furthermore, the strain exhibited considerable plasticity in its fatty acid profile in response to temperature, accumulating high levels of polyunsaturated fatty acids at 9 °C. This indicates a key adaptive strategy to its cold native habitat and underscores its potential biotechnological value. Ultimately, this study confirms that reliable species identification within *Chloroidium* requires a polyphasic approach, integrating molecular, morphological, ecological, and biochemical evidence to accurately delineate taxonomic boundaries.

## 6. Taxonomic Conclusions

*Chloroidium pseudoellipsoideum* E. Krivina, M. Sinetova & A.D. Temraleeva sp. nov.

Diagnosis—Young cells were ellipsoidal or broadly ellipsoidal, 4.5–9 × 2.1–8 µm. Chloroplasts of young cells were parietal, band-shaped to plate-shaped, containing a single pyrenoid with numerous starch granules. Adult vegetative cells were ellipsoidal, broadly ellipsoidal, or sometimes spherical, 9–16 × 5.1–16 µm. Chloroplasts of adult cells were parietal, band-, plate-, and lobed-shaped, with a single pyrenoid enveloped by numerous starch granules. The sporangia were broadly ellipsoidal or globose (8–18 × 6–16 μm). Asexual reproduction occurred via production of 2–16 unequal autospores (3–9 × 2–6 µm), released through sporangial wall rupture. Zoospores and sexual reproduction were not observed.

Differs from *C. ellipsoideum* by the absence of ovoid young cells, as well as by significant differences in cell size and autospore number. It is further distinguished by its 18S-ITS1-5.8S-ITS2 phylogeny, the presence of a CBC in helix III of ITS2, and a characteristic feature in the structure of helix IV in ITS1 and helix I in ITS2.

Holotype—Russian Federation, Sakha Republic, Duvannyi Yar, isolated from soil, in 2020, VKM Al-418cryo (metabolically inactive) deposited at the All-Russian Collection of Microorganisms (VKM), G.K. Skryabin Institute of Biochemistry and Physiology of Microorganisms, Pushchino Scientific Center for Biological Research of the Russian Academy of Sciences, Pushchino, Russian Federation.

Authentic culture—Culture of the authentic strain VKM Al-418 was deposited at the All-Russian Collection of Microorganisms (VKM), G.K. Skryabin Institute of Biochemistry and Physiology of Microorganisms, Pushchino Scientific Center for Biological Research of the Russian Academy of Sciences, Pushchino, Russian Federation. DNA sequence obtained from the authentic strain VKM Al-418 was deposited in GenBank under accession no PX026227.

Etymology—From Greek “pseudēs” (“false”) + Latin “ellipsoideum”, indicating superficial resemblance yet clear genetic distinctness from *Chloroidium ellipsoideum*.

Type locality—Russian Federation, Sakha Republic, Duvannyi Yar. The strain was isolated from soil in 2020, 68°37′51″ N 159°5′4″ E.

Note—Material of the authentic strain VKM Al-418 is maintained at the IPPAS collection of microalgae and cyanobacteria, K.A, Timiryazev Institute of Plant Physiology, Moscow, Russian Federation (under the designation IPPAS C-2091).

## Figures and Tables

**Table 1:** Morphological comparison of *Chloroidium pseudoellipsoideum* sp. nov. with other *Chloroidium* species [1,4,5,7,8,9].

Species	Young Cell			Adult Cell			Pyrenoid	Reproduction by Autospores
	Cell Shape	Cell Size, μm	Chloroplast Shape	Cell Shape	Cell Size, μm	Chloroplast Shape		
*Chloroidium* *psoromicola*	Ellipsoidal, broadly ellipsoidal, almost spherical	4.6 × 2.0– 10.5 × 6.4 (5.8 × 5.2– 9.2 × 8.5)	Parietal, band- to cup-shaped	Broadly ellipsoidal, almost spherical	6.8 × 6.7– 18.2 × 18.1	Parietal, band- to cup-shaped, deeply lobed	Naked	2–16
*Chloroidium* *saccharophilum*	Narrowly ellipsoidal, ovoid, almost cylindrical	-	Parietal, band-shaped	Ellipsoidal, almost spherical, slightly pyriform	6.9 × 5.3– 13.6 × 9.4	Parietal, band-shaped, slightly lobed	Naked	2–16
*Chloroidium* *antarcticum*	Ellipsoidal, broadly ellipsoidal, almost spherical	13.6 × 10.9– 16.4 × 11.8	Parietal, band-shaped, deeply lobed	Ellipsoidal, broadly ellipsoidal	19.1 × 16.4– 24.5 × 20.0	Parietal, deeply lobed	Naked	4–64
*Chloroidium* *arboricola*	Broadly ellipsoidal, spherical	4.1 × 2.8– 5.6 × 5.0	Parietal, band-shaped	Ellipsoidal, almost spherical	5.3 × 5.0–8.2 × 8.5 (9.2–10.0)	Parietal, band-shaped	Naked	4–8
*Chloroidium* *ellipsoideum*	Ellipsoidal, ovoid, irregular	4.4 × 2.7– 6.9 × 4.4	Parietal, plate-like, band-shaped	Ellipsoidal, broadly ellipsoidal, almost spherical	6.0 × 5.2– 10.4 × 9.2 (14.0 × 9.0)	Parietal, band-shaped, deep lobes	Present	2–8
*Chloroidium* *engadinense*	Narrowly ellipsoidal, almost cylindrical, pointed on one side	4.4 × 2.7– 7.8 × 4.2	Parietal, band-shaped	Narrowly ellipsoidal, ellipsoidal	7.6 × 4.0– 10.6 × 7.5	Parietal, band-shaped	Absent	2–8
*Chloroidium* *laureanum*	-	-	-	Spherical	(2.5–)3.0–7.5 (–9.8)	Parietal, cup-shaped, lobes	Absent	2–8
*Chloroidium* *lichinum*	Narrowly ellipsoidal, ovoid	6.0 × 3.0– 7.5 × 4.0	Ellipsoidal, ovoid, spherical	Ellipsoidal, ovoid, spherical	10.1 × 7.1– 20.8 × 9.0 (×11.5)	Parietal, deeply lobed	Present	2–8 (16)
*Chloroidium* *lobatum*	-	-	Spherical	Spherical	(3.5–)4.0–10.5 (–13.5)	Parietal, cup-shaped, often with two lobes	Absent	2–8
*Chloroidium* *orientale*	Ellipsoidal, broadly ellipsoidal	4.1 × 1.8– 7.4 × 5.1	Ellipsoidal, broadly ellipsoidal, almost spherical	Ellipsoidal, broadly ellipsoidal, almost spherical	3.8 × 2.1– 16.7 × 9.0 (5.4– 10.8)	Parietal, band- to cup-shaped	Present	2–16
*Chloroidium viscosum*	Ellipsoidal, broadly ellipsoidal	4.1 × 2.8– 5.6 × 5.0	Ellipsoidal, almost spherical	Ellipsoidal, almost spherical	6.3 × 5.0–8.8 × 6.9 (9.4–10.0)	Parietal, band-shaped	Naked	2–32
***Chloroidium pseudoellipsoideum* sp. nov.**	Ellipsoidal or broadly ellipsoidal	4.5–9 × 2.1–8	Parietal, band-shaped to plate-shaped, containing a single pyrenoid	Ellipsoidal, broadly ellipsoidal or sometimes spherical	9–16 × 5.1–16	Parietal, band-, plate- and lobed- shaped	Present	2–16
*Chloroidium* *dehydrum*	Ellipsoidal, broadly ellipsoidal, narrowly ellipsoidal, ovoid, irregular	4.3 × 3.0– 11.3 × 7.2	Parietal, ellipsoidal, ovoid, spherical	Ellipsoidal, broadly ellipsoidal, spherical	7.3 × 5.4– 18.2 × 15.3	Parietal, deeply lobed	Present	2–16
*Chloroidium* *satellitum*	Ellipsoidal, narrowly ellipsoidal, ovoid	4.1 × 3.3– 9.6 × 5.1	Parietal, ellipsoidal, broadly ellipsoidal, almost spherical	Broadly ellipsoidal, almost spherical	5.5 × 5.4– 10.7 × 10.2	Parietal, slightly lobed	Present	2–32

Note: studied strain is highlighted in bold; “-”—no information.

**Table 2:** Mantel test results.

Morphological Characteristics	Pearson Correlation Coefficient	*p*-Value
Shape of young cells	0.69	0.0001
Maximum size of young cells	0.65	0.0012
Shape of adult cells	0.62	0.0002
Pyrenoid presence	0.52	0.0001
Chloroplast type of young cells	0.52	0.0048
Number of autospores	0.35	0.0038
Chloroplast type of adult cells	0.3	0.0092
Maximum size of adult cells	0.29	0.0053

**Table 3:** Comparison of the effectiveness of different DNA barcodes for distinguishing species of the genus *Chloroidium*.

	V4 Region	V9 Region	ITS1	ITS2	ITS1-5.8S-ITS2
Barcode length, bp	430–432	175–176	244–360	211–247	591–617
Proportion of variable sites, %	4.3	15.3	62.3	59.6	52.7
Range of intraspecific distances	0–0.9	0–2.9	0–5.1	0–5.9	0–3.6
Range of interspecific distances	0–2.2	0–8.6	4.2–59.6	1.9–83.7	2.3–44.5
Percentage of overlapping distances from the total range of genetic differences, %	40.9	33.4	1.5	4.8	2.9
Efficiency of species identification, %	21.4	28.6	78.6	78.6	78.6

**Table 4:** Efficiency of species delimitation algorithms across different DNA barcodes.

		18S-ITS1-5.8S-ITS2	ITS1	ITS2	ITS1-5.8S-ITS2
ASAP	matches	90.9	90.9	81.8	81.8
	splits	9.1	9.1	18.2	18.2
KoT	matches	81.8	81.8	72.7	72.7
	splits	18.2	18.2	27.3	27.3
GMYC	matches	81.8	90.9	81.8	81.8
	splits	18.2	9.1	18.2	18.2
mlPTP	matches	81.8	72.7	81.8	72.7
	splits	18.2	27.3	18.2	27.3
bPTP	matches	81.8	81.8	72.7	72.7
	splits	18.2	18.2	27.3	27.3

**Table 5:** Fatty acid composition of strain VKM Al-418 grown at 9 °C, 22 °C, and 27 °C. Trace FAs (<0.5%) were also detected (not shown) including19:1Δ10, 20:0, 22:0, and 24:0. Data are means of three biological replications ± standard deviation. For the main fatty acids, statistical significance was assessed by one-way ANOVA with Tukey’s HSD post hoc test. Different lowercase letters within a row denote significant differences between temperatures (*p* < 0.05); letters with an asterisk denote a higher level of significance (*p* < 0.01). Abbreviations: UI, unsaturation index; n.d., not detected; and η^2^, effect size.

FAs (Mass % of Total)	VKM Al-418			η^2^
	9 °C	22 °C	27 °C	
14:0	1.0 ± 0.1	1.2 ± 0.3	1.1 ± 0.1	
16:0	20.6 ± 1.5 ^a^	23.0 ± 1.9 ^a^	41.8 ± 3.5 ^b*^	0.958
16:1 Δ7	1.9 ± 0.6	0.4 ± 0.1	0.2 ± 0.2	
16:1 Δ9	n.d.	1.0 ± 0.1	0.3 ± 0.3	
16:2 Δ7,10	1.8 ± 0.1	2.7 ± 0.1	0.4 ± 0.2	
16:3 Δ7,10,13	6.0 ± 0.9 ^a*^	1.7 ± 0.4 ^b^	0.1 ± 0.1 ^c^	0.966
18:0	2.4 ± 0.7	0.8 ± 0.6	3.4 ± 1.3	
18:1 Δ9	5.7 ± 1.3 ^a^	7.3 ± 0.1 ^a^	25.4 ± 3.0 ^b*^	0.971
18:1 Δ11	1.3 ± 0.1	2.6 ± 0.3	1.4 ± 0.1	
18:2 Δ9,12	17.1 ± 0.9 ^a^	36.1 ± 2.2 ^b*^	21.2 ± 4.0 ^a^	0.933
18:3 Δ9,12,15	41.6 ± 2.0 ^a*^	22.8 ± 1.4 ^b*^	3.0 ± 1.1 ^c*^	0.993
UI	1.892 ± 0.075 ^a^	1.623 ± 0.066 ^b^	0.802 ± 0.095 ^c*^	0.981

## Data Availability

All data analyzed in this study might be available upon request. The determined sequence of 18S-ITS1-5.8S-ITS2 region of VKM Al-418 was deposited to GenBank under accession number PX026227.

## Appendix A

**Table A1:** Genetic distances of 18S-ITS1-5.8S-ITS2 fragment between different members of the genus *Chloroidium*, %.

	*C. dehydrum*	*C. satellitum*	*C. lichinum* Group B	*C. lichinum* Group C	*C. pseudoellipsoideum* sp. nov. Group A	*C. ellipsoideum*	*C. engadinense*	*C. viscosum*	*C. psoromicola*	*C. orientale*	*C. saccharophilum*	*C. antarcticum*	*C. lobatum*	*C. laureanum*	*C. arboricola*
*C. dehydrum*	0	-	-	-	-	-	-	-	-	-	-	-	-	-	-
*C. satellitum*	2.8	0	-	-	-	-	-	-	-	-	-	-	-	-	-
*C. lichinum* Group B	2.9–3	4	0	-	-	-	-	-	-	-	-	-	-	-	-
*C. lichinum* Group C	3	3.8	0.5–0.6	0	-	-	-	-	-	-	-	-	-	-	-
*C. pseudoellipsoideum* sp. nov. Group A	3–3.1	4.2–4.3	0.8–0.9	0.7–0.8	0.1	-	-	-	-	-	-	-	-	-	-
*C. ellipsoideum*	3.4–3.5	4.4–4.5	1.1–1.2	0.9	0.7–0.8	0–0.1	-	-	-	-	-	-	-	-	-
*C. engadinense*	4.7	5.7	3.1–3.2	3	3–3.1	3.2	0	-	-	-	-	-	-	-	-
*C. viscosum*	4.7–4.8	5.8	3.1–3.2	3.3	3–3.2	3.1–3.2	1.8–1.9	0.1	-	-	-	-	-	-	-
*C. psoromicola*	4	5.5	4.3–4.4	4.4	4.1–4.2	4.3–4.4	3.1	3.2	0	-	-	-	-	-	-
*C. orientale*	4.4–4.5	5.3	3	2.9	3.1–3.2	3.4–3.5	2.1	2.6	4–4.1	0–0.4	-	-	-	-	-
*C. saccharophilum*	4.4–4.6	5.8–6	3.5–3.8	3.6–3.8	3.5–3.8	4.1–4.3	2.7–2.9	2.4–2.7	3.5–3.9	3.2–3.6	0–0.6	-	-	-	-
*C. antarcticum*	4.6	5.6	3.4	3.4	3.1–3.2	3.4–3.5	3.2	3.3	3.7	3.2–3.5	3.6–4	0	-	-	-
*C. lobatum*	7.4	8.1	6.8	6.9	6.9	6.9–7	7	6.7	5.9	7	6.5–6.9	6.5	0	-	-
*C. laureanum*	6.8	7.1	6.1–6.2	6	6.1–6.2	6.4–6.5	6.4	6.4	6	6.6–6.8	6–6.2	6.1	4.1	0	-
*C. arboricola*	7.1	7.3	6.3	6.1	6.3	6.6–6.7	6.1	6–6.1	6.1	6.1–6.3	6–6.2	6.3	4.4	4	0

## Appendix B

**Table A2:** The number and length of introns in members of the genus *Chloroidium*.

Species	Number of Introns							
	No	1	Length, n.	2	Length, n.	3	Length, n.	
*C. ellipsoideum*	-	SAG 3.95	387	SAG 2061 SAG 2142 SAG 2111 Fall/W-8	387; 379	-	-	
		CAUP H1949						
		SAG 2143						
		ISBAL-004						
		VKM Al-416						
		ISBAL-010						
		VKM Al-417						
		SAG 2140						
		ISBAL-005						
		CCAP 211/76						
		ISBAL-006						
		ISBAL-009						
		CCAP 211/101						
*C. pseudoellipsoideum*	ISBAL-008	CCAP 464/1	381	CAUP H1999-1	339; 381	-	-	
	VKM Al-418							
	SAG 2041							
*C. lichinum* Group B	SAG 2115	-	-	-	-	-		-
	CCAP 211/108							
	BII/B-9							
	MG-4							
	BII/C-1							
	SAG 2144							
*C. lichinum* Group C	-	-	-	-	-	SAG 2294	374; 335; 378	
						SAG 2293		
*C. dehydrum*	-	-	-	Edo5	342; 399	-	-	
*C. satellitum*	-	-	-	GoA2	374; 416	-	-	
*C. saccharophilum*	SAG 211-9a SAG 2055 Fall/B-1b CCAP211/42 CCAP 264/2 CCAP 211/58	SAG 211-1c	400	ISBAL-007	377; 400	-	-	
		CCAP 211/32						
		CCAP 211/40						
		CCAP 211/31						
		SAG 211-1b						
		SAG 211-1d						
		CCAP 211/27						
		SAG 2120						
		SAG 2140						
		CCAP 211/36	377					
*C. engadinense*	-	-	-	-	-	SAG 812/1	394; 368; 404	
*C. orientale*	VCA-45	-	-	-	-	-	-	
	VCA-46							
	VCA-47							
*C. viscosum*	-	-	-	MG-2 SAG 56.87	363; 401	-	-	
*C. antarcticum*	-	ISBAL-1013	422	-	-	-	-	
*C. psoromicola*	PAMC 29142	-	-	-	-	-	-	
*C. arboriculum*	-	-	-	MG-1	484; 463	-	-	
				MG-3				
*C. laureanum*	-	-	-	CAUP H8501	1280; 434	-	-	
*C. lobatum*	-	-	-	CAUP H8502	412; 466	-	-	

## Appendix C

**Table A3:** Genetic distances of ITS1 between different members of the genus *Chloroidium*, %.

	*C. dehydrum*	*C. satellitum*	*C. lichinum* Group B	*C. lichinum* Group C	*C. pseudoellipsoideum* sp. nov. Group A	*C. ellipsoideum*	*C. engadinense*	*C. viscosum*	*C. psoromicola*	*C. orientale*	*C. saccharophilum*	*C. antarcticum*	*C. lobatum*	*C. laureanum*	*C. arboricola*
*C. dehydrum*	0	-	-	-	-	-	-	-	-	-	-	-	-	-	-
*C. satellitum*	13.4	0	-	-	-	-	-	-	-	-	-	-	-	-	-
*C. lichinum* Group B	23.7	24.4	0	-	-	-	-	-	-	-	-	-	-	-	-
*C. lichinum* Group C	27.1	26.5	5.2	0	-	-	-	-	-	-	-	-	-	-	-
*C. pseudoellipsoideum* sp. nov. Group A	26.2–27.6	27.1–28.6	7.4–8.4	8.4–9.8	0–2.5	-	-	-	-	-	-	-	-	-	-
*C. ellipsoideum*	29.1	27.9	8.8–9.8	9.8–10.7	4.2–6.9	0–0.8	-	-	-	-	-	-	-	-	-
*C. engadinense*	35.4	36.4	25	23.9	21.8–23	23.5–23.6	0	-	-	-	-	-	-	-	-
*C. viscosum*	33.2–34	33.9–34.7	22–23.2	23.7–25	18.3–20.6	20–21.2	9.1–9.9	0.7	-	-	-	-	-	-	-
*C. psoromicola*	34.5	37.5	31.2	29.9	25.7–27.6	28.8	23.7	21.9	0	-	-	-	-	-	-
*C. orientale*	30.8–31.1	29.2–29.5	20.7–22.2	19.6–21.2	21.1–23.9	23.5–26.3	20.8–23.5	20.4–23.2	33.4–34.3	0–3.9	-	-	-	-	-
*C. saccharophilum*	27.3–30.8	30.7–33	18.9–21.8	21.9–24.9	17.6–21.1	21.8–23.6	12.7–14.9	13–15.7	25.6–27.8	24.4–27	0–5.1	-	-	-	-
*C. antarcticum*	33.3	38.1	18.9	19.5	17.6–18.8	20.4–21.5	17.9	17.8–18.9	21.7–23.2	20.5–21.7	22.1–24.5	0	-	-	-
*C. lobatum*	59.5	58.9	54.3	53	51.3	49.5	53.2	49.9	45	51.9–52.4	48–51	53.6	0	-	-
*C. laureanum*	46.7	46.7	39.1	40	40.4–42.7	42–43.5	47.8	45.4	46.9	44.5–45	42.6–44.8	47	43.6	0	-
*C. arboricola*	55	54.6	42.6	40	42–43.5	46–46.8	49.6	46.6–48.3	49.9	40.1–43.7	38.4–42	44.7	44.7	41.8	0

## Appendix D

**Table A4:** Genetic distances of ITS2 between different members of the genus *Chloroidium*, %.

	*C. dehydrum*	*C. satellitum*	*C. lichinum* Group B	*C. lichinum* Group C	*C. pseudoellipsoideum* sp. nov. Group A	*C. ellipsoideum*	*C. engadinense*	*C. viscosum*	*C. psoromicola*	*C. orientale*	*C. saccharophilum*	*C. antarcticum*	*C. lobatum*	*C. laureanum*	*C. arboricola*
*C. dehydrum*	0	-	-	-	-	-	-	-	-	-	-	-	-	-	-
*C. satellitum*	27.3	0	-	-	-	-	-	-	-	-	-	-	-	-	-
*C. lichinum* Group B	28.5	48	0	-	-	-	-	-	-	-	-	-	-	-	-
*C. lichinum* Group C	31	47.7	4.4	0	-	-	-	-	-	-	-	-	-	-	-
*C. pseudoellipsoideum* sp. nov. Group A	28.1	44	3.4	5.1	0–0.2	-	-	-	-	-	-	-	-	-	-
*C. ellipsoideum*	30.9–31.8	45.2–46.5	4.4–4.9	6.2–6.7	1.9–2.4	0–0.5	-	-	-	-	-	-	-	-	-
*C. engadinense*	53	62.5	33.3	32.1	31.8	33.5–34.4	0	-	-	-	-	-	-	-	-
*C. viscosum*	48.4–49.6	59.7–61.2	28.9–29	30–30.1	26.8	28.3–29.1	15.8	1.7	-	-	-	-	-	-	-
*C. psoromicola*	32.3	42.6	44.8	47.1	46.5	47.7–49	36.5	39.3–40.4	0	-	-	-	-	-	-
*C. orientale*	48.6–49.8	56.9–63.2	24.5–26	23.7–25.1	23.5–24.7	24.6–26.9	15.1–16.1	18.9–19.5	41.1–43.3	0–5.9	-	-	-	-	-
*C. saccharophilum*	42.8–45	61.6–64.7	33.3–36	32.2–35	30.8–33.5	32.6–38.1	37.7–41.9	32.7–37.9	42.6–44.9	38.3–41.3	0–4	-	-	-	-
*C. antarcticum*	47.1	60.2	35.6	33.8	38.6	36.7–37.6	49.5	47.5–47.6	55.3	36.9–43.4	40.3–42.2	0	-	-	-
*C. lobatum*	57.3	67.8	52.2	52.7	54	54–55.2	83.7	72.7	59.8	70.7–71	64.1–68	57.9	0	-	-
*C. laureanum*	41.5	55.9	39.9	39.7	38.3	42.1	61.1	59.5–60.9	52.2	53.1–56.4	45.1–48.1	46	30.4	0	-
*C. arboricola*	64.1	68.1	54.2	51	56.5	59.1	58.5	57.4–60.8	60.6	52.8–57.7	55.2–56.4	57	43	28.3	0

## Appendix E

**Table A5:** Genetic distances of ITS1-5.8S-ITS2 fragment between different members of the genus *Chloroidium*, %.

	*C. dehydrum*	*C. satellitum*	*C. lichinum* Group B	*C. lichinum* Group C	*C. pseudoellipsoideum* sp. nov. Group A	*C. ellipsoideum*	*C. engadinense*	*C. viscosum*	*C. psoromicola*	*C. orientale*	*C. saccharophilum*	*C. antarcticum*	*C. lobatum*	*C. laureanum*	*C. arboricola*
*C. dehydrum*	0	-	-	-	-	-	-	-	-	-	-	-	-	-	-
*C. satellitum*	14.1	0	-	-	-	-	-	-	-	-	-	-	-	-	-
*C. lichinum* Group B	17.8	22.6	0	-	-	-	-	-	-	-	-	-	-	-	-
*C. lichinum* Group C	20	23.1	3.7	0	-	-	-	-	-	-	-	-	-	-	-
*C. pseudoellipsoideum* sp. nov. Group A	18.6–19.1	23.1–23.7	4.4–4.8	5.6–6.1	0–1	–	–	–	–	–	–	–	–	–	–
*C. ellipsoideum*	20.4–20.6	23.7–24	5.3–5.7	6.5–6.8	2.3–3.4	0–0.5	–	–	–	–	–	–	–	–	–
*C. engadinense*	29	31	20.8	20.2	19.1–19.6	20.3–20.5	0	–	–	–	–	–	–	–	–
*C. viscosum*	27.2–27.8	30	18.4–18.8	19.6–20	16.4–17.2	17.5–18.1	9.4–9.7	0.9	–	–	–	–	–	–	–
*C. psoromicola*	24.1	27.8	25.4	25.8	23.6–24.3	25.1–25.4	20	20.4–20.6	0	–	–	–	–	–	–
*C. orientale*	26–26.3	27–28.1	16.4–17.4	16.3–16.5	16.6–18.1	17.9–19.4	14.3–14.9	14.8–15.7	25.9–26	0–3.6	–	–	–	–	–
*C. saccharophilum*	23.4–25.2	27.9–29.6	18.1–19.3	19.2–20.4	17.6–18.8	20–21	17.6–18.8	16.1–17.8	23–24.2	21.9–22.8	0–3.2	–	–	–	–
*C. antarcticum*	26.3	31.3	18.9	18.9	18.5–19	19.2–19.9	21.9	21.5–21.9	25.4	20.7–21.9	21.8–22.7	0	–	–	–
*C. lobatum*	38.6	41.3	36.4	36.5	35.6–36.1	35.2–35.5	44.5	41.1	36.8	41–41.2	37.2–38.9	39.1	0	–	–
*C. laureanum*	30.4	33.4	27.5	27.5	27.4–28.1	29–29.5	36.5	35.3–35.6	34.9	33.8–34.4	30.6–31.9	33.3	29	0	–
*C. arboricola*	38.2	37.9	31.9	30.1	32.8–33.3	34.7–35	36.7	35.4–36.8	37.7	32.7–32.9	31.3–32.6	35	32.5	26.4	0

## Appendix F

**Table A6:** Results of overlap minimizing and ROC analysis.

Parameter	18S-ITS1-5.8S-ITS2	ITS1	ITS2	ITS1-5.8S-ITS2
Maximum intraspecific distance, %	0.6	5.1	5.9	3.6
Minimum interspecific distance, %	0.70	4.2	1.9	2.3
Overlap minimizing threshold, %	0.65	4.65	3.9	2.95 ≈ 3.0
Area of distribution overlap, %	≈2–3	≈10	≈40–45	≈15–20
Optimal ROC Threshold, %	0.65	4.6	3.8	3.0
Area Under Curve (AUC)	0.982	0.992	0.973	0.987
95% CI (AUC)	0.94–1.00	0.96–1.00	0.93–1.00	0.95–1.00
Sensitivity	1.00	0.99	0.96	0.98
Specificity	0.98	0.99	0.95	0.97
Accuracy	0.99	0.99	0.96	0.98
